# Features of *TP53*-mutated patients with chronic myelomonocytic leukemia in a national (ABCMML) and international cohort (cBIOPORTAL)

**DOI:** 10.1007/s10354-025-01072-0

**Published:** 2025-03-05

**Authors:** Magdalena Grass, Klaus Geissler

**Affiliations:** Medical School, Sigmund Freud University, Freudplatz 3, 1020 Vienna, Austria

**Keywords:** CMML, TP53, ABCMML, cBIOPORTAL, Mutations, CMML, TP53, ABCMML, cBIOPORTAL, Mutationen

## Abstract

Big data collected in large international cooperations allow validation of findings from traditional national patient cohorts for proving consistency. In this study we analyzed outcomes and phenotypic features of *TP53*-mutated chronic myelomonocytic leukemia (CMML) patients in the Austrian biodatabase for CMML (ABCMML; *n* = 322) and in the international platform cBIOPORTAL (*n* = 399). The prevalences of *TP53* mutations were 1.58 and 3.66, respectively. Numerically, overall survival was shorter in *TP53*-mutated patients in both cohorts (ABCMML 10.0 vs. 30.0 months and cBIOPORTAL 8.9 vs. 34.5 months), but this was statistically significant only in the cBIOPORTAL cohort. Decreased hemoglobin values and the presence of blast cells in peripheral blood were significantly associated with *TP53* mutations in the cBIOPORTAL group but not in the ABCMML database. Our study indicates the necessity of sufficient patient numbers for the comparison of CMML patients regarding outcome and phenotype according to their molecular subtype, particularly in the case of rare mutations.

**Supplementary Information:**

The online version of this article (10.1007/s10354-025-01072-0) contains supplementary material, which is available to authorized users.

## Abstract (de)

Große Datenmengen aus internationalen Kooperationen ermöglichen die Validierung nationaler Patientenkohorten zur Überprüfung der Konsistenz von Daten. In dieser Studie verglichen wir Verlauf und phänotypische Eigenschaften *TP53*-mutierter Patienten der nationalen CMML(„chronic myelomonocytic leukemia“)-Biodatenbank, Austrian Biodatabase for chronic myelomonocytic leukemia, (ABCMML, *n* = 322) und der internationalen Plattform cBIOPORTAL (*n* = 399). Die Prävalenzen der *TP53* Mutationen betrugen 1,58 bzw. 3,66 %. In beiden Kohorten war das Gesamtüberleben *TP53*-mutierter Patienten kürzer als das von Wildtyppatienten (ABCMML 10,0 vs. 30,0 Monate, cBIOPORTAL 8,9 vs. 34,5 Monate), der Unterschied war aber nur in der cBIOPORTAL-Kohorte signifikant. Ein vermindertes Hämoglobin und das Vorhandensein zirkulierender Blasten waren in der cBIOPORTAL-Kohorte mit *TP53* Mutationen signifikant assoziiert, nicht aber in der ABCMML-Kohorte. Unsere Studie zeigt, dass für den Vergleich von CMML-Patienten hinsichtlich Outcome und Phänotyp nach ihrem molekularen Subtyp eine ausreichende Fallzahl notwendig ist, vor allem bei seltenen Mutationen.

## Introduction

Chronic myelomonocytic leukemia (CMML) is a rare, phenotypically and genetically diverse hematologic cancer that affects the elderly and has an inherent risk of developing into secondary acute myeloid leukemia (AML). According to the French American British (FAB) criteria, CMML was initially separated into two groups based on the presence of myeloproliferation: myeloproliferative disorder (MP-CMML; WBC count > 13 × 10^9^/L) and myelodysplastic syndrome (MD-CMML; WBC count < 13 × 10^9^/L) [1, 2]. The World Health Organization (WHO) classified CMML as belonging to the mixed category MDS/MPN in 2002 because it possesses characteristics of both an MDS and an MPN [3]. Two groups recently reported updated diagnostic criteria for CMML following the 2016 revision to the World Health Organization’s classification of myeloid neoplasms and acute leukemia [4–6]. The outcome of CMML patients can vary greatly, indicating that a number of factors may influence how the disease progresses and what causes these patients to die [7–13].

The Austrian Biodatabase for CMML (ABCMML) was recently reported. Patients with CMML have had their epidemiologic, hematologic, biochemical, clinical, immunophenotypic, cytogenetic, molecular, and biologic data gathered from various Austrian centers for 40 years [14]. It has been demonstrated to be a representative and practical source of real-world data for biomedical research.

Because of the molecular heterogeneity of CMML, it is critical to understand the meaning of molecular characteristics so that the patient can be provided with the best care possible for their unique circumstances. The effects of molecular aberrations on the clinical outcome and phenotype of disease have been examined in a few studies, but the majority of these studies’ conclusions were not confirmed by separate cohorts. However, until a prognostic parameter’s usefulness has been established, it should not be used in clinical settings. Evaluation of a prognostic parameter’s performance in a sample different from the one used to build the model is known as external validation [15].

Big data containing a huge number of datasets from large international consortium efforts are now available for many cancer entities including CMML. The cBIOPORTAL platform is such a collection of big data aiming to build a platform to support clinical decisions for personalized cancer treatment [16]. Moreover, due to the large number of well-characterized patients, it is a perfect source of data for validation of findings in traditional, sometimes much smaller patient cohorts. In this study we used data from CMML patients documented in cBIOPORTAL to validate the features of *TP53*-mutated CMML patients who have been analyzed in the ABCMML.

## Patients and methods

### Patients

#### ABCMML analysis

The ABCMML can serve as a representative and practical real-world data source for biomedical research, as we have recently demonstrated [14]. Epidemiologic, hematologic, biochemical, clinical, immunophenotypic, cytogenetic, molecular, and biologic data of CMML patients from various centers were gathered retrospectively and included in this database. Leukemic transformation and CMML were diagnosed based on WHO criteria [2–4]. Patient records were used to gather routine laboratory and clinical parameters. Prior to analyzing data from institutions, a thorough central manual retrospective chart review was conducted to guarantee data quality. This study did not include CMML patients who were undergoing transformation. Overall survival (OS), acute myeloid leukemia (AML)-free survival, and phenotypic parameter differences between mutated and wildtype patients were all analyzed using mutation data from 327 patients. On June 10, 2015, the City of Vienna’s ethics committee approved this study (ethic code: 15-059-VK).

#### cBIOPORTAL analysis

The cBIOPORTAL for Cancer Genomics provides visualization, analysis, and download of large-scale cancer genomics datasets [16]. We selected the myelodysplastic syndromes (MDS IWG, IPS SM, NEJM Evidence 2022) dataset containing 399 CMML cases with data including age, sex, white blood cell count (WBC), hemoglobin (Hb), platelets, OS, AML-free survival, bone marrow (BM) blasts, circulating blasts, cytogenetics, and gene mutations (http://www.cbioportal.org) to analyze OS, AML-free survival, and differences in phenotypic parameters between mutated and nonmutated patients.

### Statistical analysis

To ascertain whether individual parameters were connected to OS, the log-rank test was employed. OS was defined as either the last follow-up (censored) or the time from sampling to death (uncensored). The time between sampling and either transformation into AML or death (uncensored) or the last follow-up (censored) was referred to as the AML-free survival. The chi-squared test was used to compare dichotomous variables between groups. When continuous variables were not normally distributed, two unmatched groups were compared using the Mann–Whitney U test. At *p* < 0.05, the results were deemed significant. SPSS v. 27 (IBM Corp., Armonk, NY, USA) was used for statistical analyses; two-sided *p*-values were reported. Mutations with a variant allele frequency (VAF) of at least 5% in the ABCMML database and at least 2% in the cBIOPORTAL platform are regarded as positive.

## Results

### Characteristics of patients and prevalences of *TP53* mutations

The baseline characteristics of both CMML cohorts are shown in supplementary tables 1 and 2. Analyzed were 327 patients in the ABCMML cohort and 399 patients in the cBIOPORTAL cohort. As seen in other CMML series, in both cohorts there were more males than females and more than half of the patients were aged 70 years or older [13]. All characteristics except leukocytes were comparable between the cohorts. The proportion of patients with leukocytes > 13 G/L was significantly higher in the ABCMML cohort as compared to the cBIOPORTAL cohort (57% vs. 32%, *p* < 0.001). The median leukocyte counts were 14.1 vs. 9.2 G/L in these cohorts, respectively. Regarding clinical outcome, the median survival was 29.0 months in the ABCMML cohort as compared to 31.6 months in the cBIOPORTAL cohort. The prevalences of *TP53* mutations were 1.58 (5/316) in the ABCMML group and 3.66 (14/383) in the cBIOPORTAL group.

**Table 1:** Phenotypic features of ABCMML patients including leukocytosis, anemia, thrombocytopenia, and circulating blasts stratified by the presence or absence of *TP53* mutation

Parameter	With *TP53* mutation	Without *TP53* mutation	*P*-value
WBC ≥ 13 G/L	4/5 (80%)	148/311 (48%)	0.199
Hb < 10 g/dL	2/5 (40%)	213/311 (32%)	0.653
PLT < 100 G/L	3/5 (60%)	133/312 (43%)	0.655
PB blasts present	1/4 (25%)	60/261 (23%)	1.000

*WBC* white blood cell count, *Hb* hemoglobin, *PLT* platelets, *PB* peripheral blood

**Table 2:** Phenotypic features of cBIOPORTAL patients including leukocytosis, anemia, thrombocytopenia, and circulating blasts stratified by the presence or absence of *TP53* mutation

Parameter	With *TP53* mutation	Without *TP53* mutation	*P*-value
WBC ≥ 13 G/L	3/14 (21%)	118/369 (32%)	0.562
Hb < 10 g/dL	11/14 (79%)	136/383 (36%)	0.003
PLT < 100 G/L	9/14 (64%)	146/378 (39%)	0.091
PB blasts present	9/12 (75%)	79/321 (25%)	<0.001

*WBC* white blood cell count, *Hb* hemoglobin, *PLT* platelets, *PB* peripheral blood

### Impact of *TP53* mutations on survival and AML-free survival

**Fig. 1:**
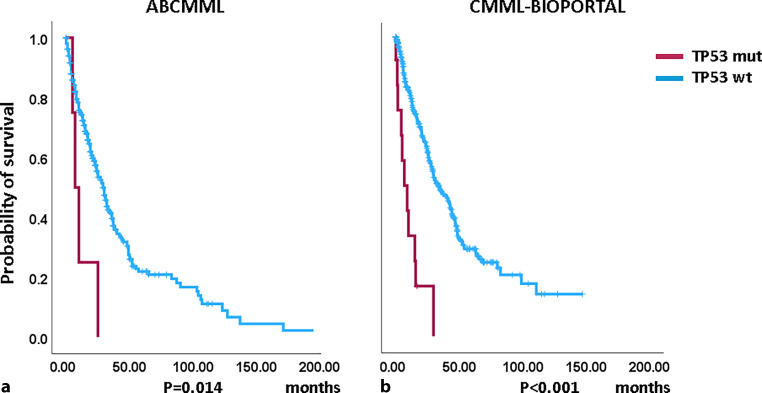
Kaplan-Meier plots for overall survival in CMML patients (**a**) from the ABCMML cohort and (**b**) the cBIOPORTAL cohort with and without *TP53* mutations. *mut* mutated, *wt* wildtype

**Fig. 2:**
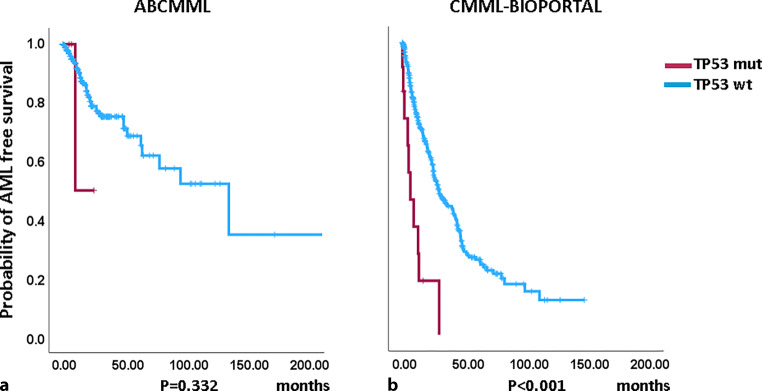
Kaplan-Meier plots for overall survival in CMML patients (**a**) from the ABCMML cohort and (**b**) the cBIOPORTAL cohort with and without *TP53* mutations. *mut* mutated, *wt* wildtype

Figures 1 and 2 show the Kaplan–Meier curves of OS in *TP53*-mutated (variants and variant allele frequencies are shown in supplementary tables 3 and 4) and *TP53*-nonmutated patients in both cohorts. In the cBIOPORTAL cohort, *TP53*-mutated patients had significantly inferior survival and AML-free survival as compared to nonmutated patients. In the ABCMML cohort, survival and AML-free survival were numerically shorter in *TP53*-mutated patients, but this did not reach significance. The median survival of *TP53*-mutated patients was 10.0 vs. 30.0 months (*p* = 0.195) in the ABCMML patients and 8.9 vs. 34.5 (*p* < 0.001) months in the cBIOPORTAL patients. The median AML-free survival was 49.0 vs. 134.0 (*p* = 0.125) in the ABCMML cohort and 6.4 vs. 29.2 (*p* < 0.001) months, respectively, in the cBIOPORTAL cohort.

### Laboratory features in the presence or absence of *TP53* mutations

**Fig. 3:**
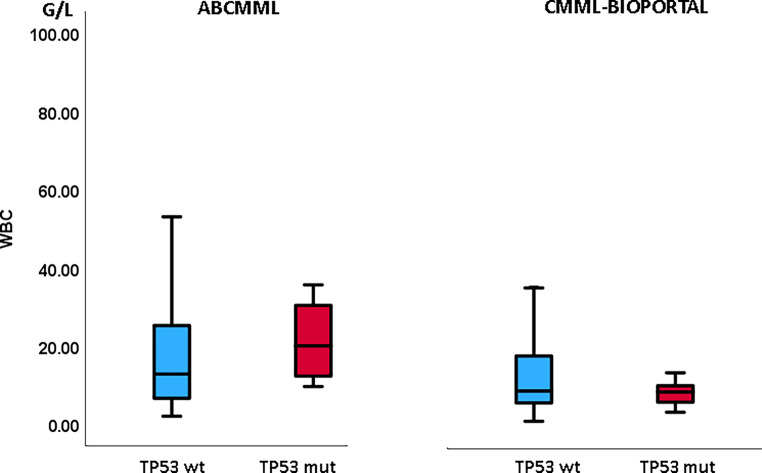
Boxplots showing the distribution of leukocytes in *TP53*-wildtype and *TP53*-mutated CMML patients including median values, minimum values, maximum values, and upper and lower quartiles in both study cohorts. *mut* mutated, *wt* wildtype

**Fig. 4:**
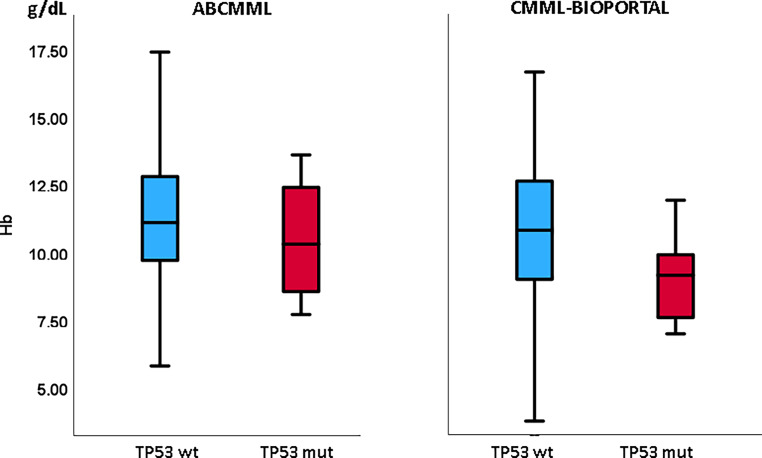
Boxplots showing the distribution of hemoglobin values in *TP53*-wildtype and *TP53*-mutated CMML patients including median values, minimum values, maximum values, and upper and lower quartiles in both study cohorts. *mut* mutated, *wt* wildtype

**Fig. 5:**
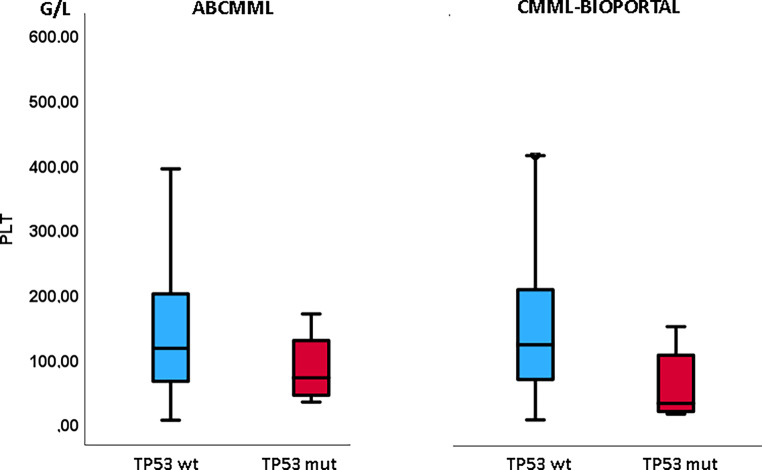
Boxplots showing the distribution of platelets in *TP53*-wildtype and *TP53*-mutated CMML patients including median values, minimum values, maximum values, and upper and lower quartiles in both study cohorts. *mut* mutated, *wt* wildtype

Tables 1 and 2 show the phenotypic parameters in the ABCMML and the cBIOPORTAL patients, respectively. In both cohorts, *TP53*-mutated patients had a significantly higher proportion of patients with leukocytosis > 13 G/L and of patients with circulating blasts. Moreover, in both cohorts, *TP53*-mutated patients had a significantly higher proportion of patients with thrombocytopenia, whereas the proportion of patients with anemia was not different. In Figs. 3, 4, and 5, metric values are visualized by boxplot diagrams. In the ABCMML cohort, the median values of *TP53*-mutated and nonmutated patients were for WBC 15.0 vs. 12.8 G/L, for Hb 10.2 vs. 11.1 g/dL, and for platelets 87 vs. 117 G/L, respectively. In the cBIOPORTAL cohort, the median values of *TP53*-mutated and nonmutated patients were for WBC 9.0 vs. 9.3 G/L, for Hb 9.2 vs. 10.8 g/dL, and for platelets 33 vs. 123 G/L, respectively.

## Discussion

In this study we analyzed a national CMML cohort from Austria (ABCMML) and an international cohort of CMML patients (cBIOPORTAL) regarding clinical, epidemiologic, and hematologic features of *TP53*-mutated patients in order to get information on the consistency and general validity of findings.

Five to ten percent of cases of AML and de novo myelodysplastic syndrome (MDS) have *TP53* mutations [17]. In contrast, up to 30–40% of patients with therapy-related MDS and AML have *TP53* mutations. Missense mutations found in a few common hotspots account for the majority of inactivating mutations seen in MDS and AML. A loss-of-function effect on normal p53 function is determined by *TP53* missense mutations in conjunction with truncating mutations or chromosomal loss of *TP53*. The selection pressure of chemotherapy or MDM2 inhibitor therapy causes *TP53*-mutant clones to grow clonally. Current chemotherapy is ineffective against *TP53*-mutant clones. A complex karyotype and a generally poor prognosis are linked to *TP53* mutations.

There are some studies analyzing the prevalence and role of *TP53* mutations in CMML. The prevalence of these mutations has a wide range, from 1–8.3%. In one Chinese study of CMML patients, the prevalence of *TP53* mutations was 8.3% [13]. On the other hand, the prevalence was 1% in other series [18]. In our study, the prevalence of *TP53* mutations was 1.6% in the ABCMML cohort and 3.7% in the cBIOPORTAL cohort. Differences regarding the prevalence of *TP53* mutations may partly be due to different proportions of therapy-related CMML cases in reported series. In an American study the prevalence of molecular aberrations of *TP53* was 4.3% in de novo CMML and 11.8% in therapy-related CMML [19]. Interestingly, this group also evaluated a cohort of 189 patients with CMML-associated AML [20]. They found that transformation occurs through distinct trajectories characterized by genomic profiles and clonal evolution: monocytic (Mo-AML, 53%), immature myeloid (My-AML, 43%), or erythroid AML (Ery-AML, 2%), which were defined by complex karyotypes and *TP53* mutations.

There is only limited information on the clinical outcome of *TP53* mutations in CMML series. In a study reported by Gurney, 31/1315 (2.4%) patients had concurrent *TP53* alterations. There was a highly significant inferior impact of *TP53* alteration in this study [21]. In another study of the effect of *TP53* mutation variant allele frequency on phenotype and outcomes in myelodysplastic syndromes, only *TP53* mutations with VAF  40% were an independent factor for inferior survival in multivariate analysis [22]. In the cBIOPORTAL cohort, *TP53*-mutated patients had significantly inferior survival and AML-free survival as compared to nonmutated patients. In the ABCMML cohort, survival and AML-free survival were numerically shorter in *TP53*-mutated patients, but this did not reach significance. Looking at the median survival numbers, the shape of the Kaplan–Meier curves, and the low number of mutated patients, it is likely that the results would have reached significance with higher patient numbers, indicating the necessity of sufficient patient numbers for the comparison of CMML patients regarding outcome and phenotype according to their molecular subtype, particularly in the case of rare mutations.

The correlation of phenotypic features with the mutational status in CMML patients has been described previously [18], but regarding *TP53* there are only limited data. In our study decreased platelet counts, decreased Hb values, and the presence of blast cells in PB were significantly associated with *TP53* mutations in the cBIOPORTAL cohort but not in the ABCMML cohort.

Limitations of this study include the fact that the proportion of patients with leukocytes  13 G/L was significantly higher in the ABCMML cohort as compared to the cBIOPORTAL cohort. The reason for this imbalance is not completely clear. Increased laboratory screening in recent years in asymptomatic persons may detect some diseases including CMML in an earlier phase than in the past. Therefore, older patient series may be enriched in patients with more advanced disease as compared to more recent series. In fact, we have seen a significant drop in the proportion of patients with MP-CMML from 66% to 48% since 2010 in the ABCMML database (unpublished data).

Changes in the diagnostic criteria of CMML over time since its first description in 1982 represent another limitation of the ABCMML database, suggesting that this patient group is more heterogenous as compared to the cBIOPORTAL group which contains patients that were included over a shorter period of time. Furthermore, it needs to be considered that a proportion of patients in ABCMML, in particular older patients, did not consent to have BM puncture. However, we do not think that this greatly affected diagnostic accuracy, since persistent peripheral blood monocytosis is the most important diagnostic feature, and a genoclinical model has been recently described that uses mutational data, peripheral blood values, and clinical variables to predict the MDS vs. CMML diagnosis with high accuracy in the absence of a BM biopsy result [23]. Moreover, somatic mutations associated with CMML were not only detected in CMML patients confirmed by BM biopsy but also in 57% of patients with nondiagnostic BM features. Interestingly, the OS in non-diagnostic-mutated patients was indistinguishable from CMML, suggesting that the mutational spectrum is a much more sensitive parameter for the detection of myeloid malignancies as compared to BM morphology [24].

In recent years, health care management has changed from a disease-centered model to a patient-centered model [25]. The adoption of big data characterized by a large amount of digital data which are continually generated by people within clinical care and everyday life will enable implementation of personalized and precise medicine based on personalized information. Moreover, these data can be used for validation of findings from national cohorts, as we have shown in this study, and thus make an important contribution to optimizing patient management.

## Supplementary Information

*Suppl Table 1:* Patient characteristics in the ABCMML cohort

*Suppl Table 2***:** Patient characteristics in the CMML cBIOPORTAL cohort

*Suppl Table 3***:** *TP53* variants and variant allele frequencies in patients of the ABCMML cohort

*Suppl Table 4***:** *TP53* variants and variant allele frequencies in patients of the cBIOPORTAL cohort
