# Supplementary material for: Features of TP53-mutated patients with chronic myelomonocytic leukemia in a national (ABCMML) and international cohort (cBIOPORTAL)
Source: Wien Med Wochenschr. 2025 Mar 5;175(11-12):302–8. doi: 10.1007/s10354-025-01072-0 (PMC12380936; doi:10.1007/s10354-025-01072-0)
Supplement: Supplementary file 1 — Suppl Table 1: Patient characteristics in the ABCMML cohort [file 10354_2025_1072_MOESM1_ESM.docx]

**Suppl Table 1:** Patient characteristics in the ABCMML cohort

|  | Cases  N=327 | Percent |
| --- | --- | --- |
| Age  Evaluable = 327 |  |  |
| <70 years | 114 | 35% |
| >70 years | 213 | 65% |
| Sex  Evaluable = 327 |  |  |
| Male | 204 | 62% |
| Female | 123 | 38% |
| Leukocytes  Evaluable = 316 |  |  |
| >13 G/L | 152 | 48% |
| <13 G/L | 164 | 52% |
| Hemoglobin  Evaluable = 316 |  |  |
| <10 g/dL | 100 | 32% |
| >10 g/dL | 216 | 68% |
| Platelets  Evaluable = 317 |  |  |
| <100 G/L | 136 | 43% |
| >100 G/L | 181 | 57% |
| PB Blasts  Evaluable = 265 |  |  |
| absent | 204 | 77% |
| present | 61 | 23% |
