# Supplementary material for: Features of TP53-mutated patients with chronic myelomonocytic leukemia in a national (ABCMML) and international cohort (cBIOPORTAL)
Source: Wien Med Wochenschr. 2025 Mar 5;175(11-12):302–8. doi: 10.1007/s10354-025-01072-0 (PMC12380936; doi:10.1007/s10354-025-01072-0)
Supplement: Supplementary file 2 — Suppl Table 2: Patient characteristics in the CMML cBIOPORTAL cohort [file 10354_2025_1072_MOESM2_ESM.docx]

**Suppl Table 2:** Patient characteristics in the CMML-BIOPORTAL cohort

|  | Cases  N=399 | Percent |
| --- | --- | --- |
| Age  Evaluable = 399 |  |  |
| <70 years | 136 | 34% |
| >70 years | 263 | 66% |
| Sex  Evaluable = 399 |  |  |
| Male | 268 | 67% |
| Female | 130 | 33% |
| Leukocytes  Evaluable = 383 |  |  |
| >13 G/L | 121 | 32% |
| <13 G/L | 262 | 68% |
| Hemoglobin  Evaluable = 397 |  |  |
| <10 g/dL | 147 | 37% |
| >10 g/dL | 250 | 63% |
| Platelets  Evaluable = 392 |  |  |
| <100 G/L | 155 | 40% |
| >100 G/L | 237 | 60% |
| PB Blasts  Evaluable = 333 |  |  |
| absent | 245 | 74% |
| present | 88 | 26% |
