# Supplementary material for: Features of TP53-mutated patients with chronic myelomonocytic leukemia in a national (ABCMML) and international cohort (cBIOPORTAL)
Source: Wien Med Wochenschr. 2025 Mar 5;175(11-12):302–8. doi: 10.1007/s10354-025-01072-0 (PMC12380936; doi:10.1007/s10354-025-01072-0)
Supplement: Supplementary file 3 — Suppl Table 3: TP53 variants and variant allele frequencies in patients of the ABCMML cohort [file 10354_2025_1072_MOESM3_ESM.docx]

**Suppl Table 3:** *TP53* variants and variant allele frequencies in patients of the ABCMML

| **ABCMML ID** | **TP53** | **VAF** |
| --- | --- | --- |
| CMML_1_027 | R175C | 35 |
| CMML_1_632 | V73fs | 48 |
| CMML_16_372 | A161T | 53 |
| CMML_1_470 | G105D | 13 |
| CMML_18_678 | R248W | 45 |
