# Supplementary material for: Features of TP53-mutated patients with chronic myelomonocytic leukemia in a national (ABCMML) and international cohort (cBIOPORTAL)
Source: Wien Med Wochenschr. 2025 Mar 5;175(11-12):302–8. doi: 10.1007/s10354-025-01072-0 (PMC12380936; doi:10.1007/s10354-025-01072-0)
Supplement: Supplementary file 4 — Suppl Table 4: TP53 variants and variant allele frequencies in patients of the cBIOPORTAL cohort [file 10354_2025_1072_MOESM4_ESM.docx]

**Suppl Table 4:** *TP53* variants and variant allele frequencies in patients of the BIOPORTAL

| **BIOPORTAL ID** | **TP53** | **VAF** |
| --- | --- | --- |
| E-H-100088 | D228V*18, N311T*34 | 29 |
| E-H-100221 | V272M | 49 |
| E-H-103088 | P177_c182del | 42 |
| E-H-103094 | R248W | 80 |
| E-H-105485 | C135Y | 92 |
| E-H-105531 | M237I, X125_splice | 47 |
| E-H-116406 | H193L | 17 |
| E-H-116603 | H193R | 23 |
| E-H-116720 | L330P, X125_splice | 45 |
| E-H-117173 | Y163C, N131I | 39 |
| E-H-117992 | R306* | 79 |
| E-H-118249 | R248Q | 43 |
| E-H-118399 | V272M, T125R | 51 |
| E-H-118417 | R175H | 53 |
